# Multimodal physiological correlates of surgeon stress in live robot-assisted surgery

**DOI:** 10.1007/s00464-026-13054-3

**Published:** 2026-06-30

**Authors:** Kaiqi Wei, Nanako Nakamura, Megumi Shimura, Yoshihiro Shimomura, Xue Zhao, Takaaki Tamura, Shinichi Sakamoto

**Affiliations:** 1 Department of Design, Division of Creative Engineering, Graduate School of Science and Engineering, Chiba University, Chiba, Japan; 2 Design Research Institute, Chiba University, 1-33 Yayoi-cho, Inage-ku, Chiba, 263-8522 Japan; 3 Department of Urology, Graduate School of Medicine, Chiba University, Chiba, Japan

**Keywords:** Robot-assisted surgery, Surgeon stress, Intraoperative assessment, Physiological monitoring, Video-stimulated recall

## Abstract

**Background:**

Robot-assisted surgery (RAS) imposes cognitive and psychomotor demands on console surgeons, but the physiological correlates of perceived stress during live procedures remain insufficiently characterized. Prior work has often relied on simulations or broad phase-based assessments, providing limited insight into short-term stress fluctuations in the operating room.

**Methods:**

We conducted a prospective observational field study during live RAS procedures. Seven senior urologists were monitored during one procedure each using wireless electroencephalography (EEG), surface electromyography (sEMG), and electrocardiography (ECG). ECG-derived heart rate variability (HRV) was calculated. To avoid disrupting surgical workflow, surgeons retrospectively annotated perceived stress using video-stimulated recall (VSR) of console-view videos. Associations between physiological features and stress ratings were assessed using a linear mixed-effects model.

**Results:**

Across 151 VSR-based annotation windows, higher perceived stress was associated with a higher beta-to-alpha power ratio at the central midline EEG channel (Cz; *β* = 0.42, 95% confidence interval [CI] 0.08 to 0.75), greater upper trapezius sEMG activity (*β* = 0.37, 95% CI 0.06 to 0.68), shorter mean normal-to-normal cardiac intervals (Mean NN; *β* = − 0.39, 95% CI − 0.70 to − 0.07), and lower standard deviation of normal-to-normal intervals (SDNN; *β* = − 0.44, 95% CI − 0.86 to − 0.01). These directions were generally consistent across robustness and sensitivity analyses. Other EEG spectral and HRV features were not significantly associated with perceived stress.

**Conclusions:**

In live RAS, perceived stress reported by console surgeons was associated with selected EEG, sEMG, and HRV features. These findings suggest that combining VSR-based stress annotations with limited physiological monitoring is feasible during live RAS and may help identify high-demand operative moments during postoperative review.

**Graphical abstract:**

**Figure:**
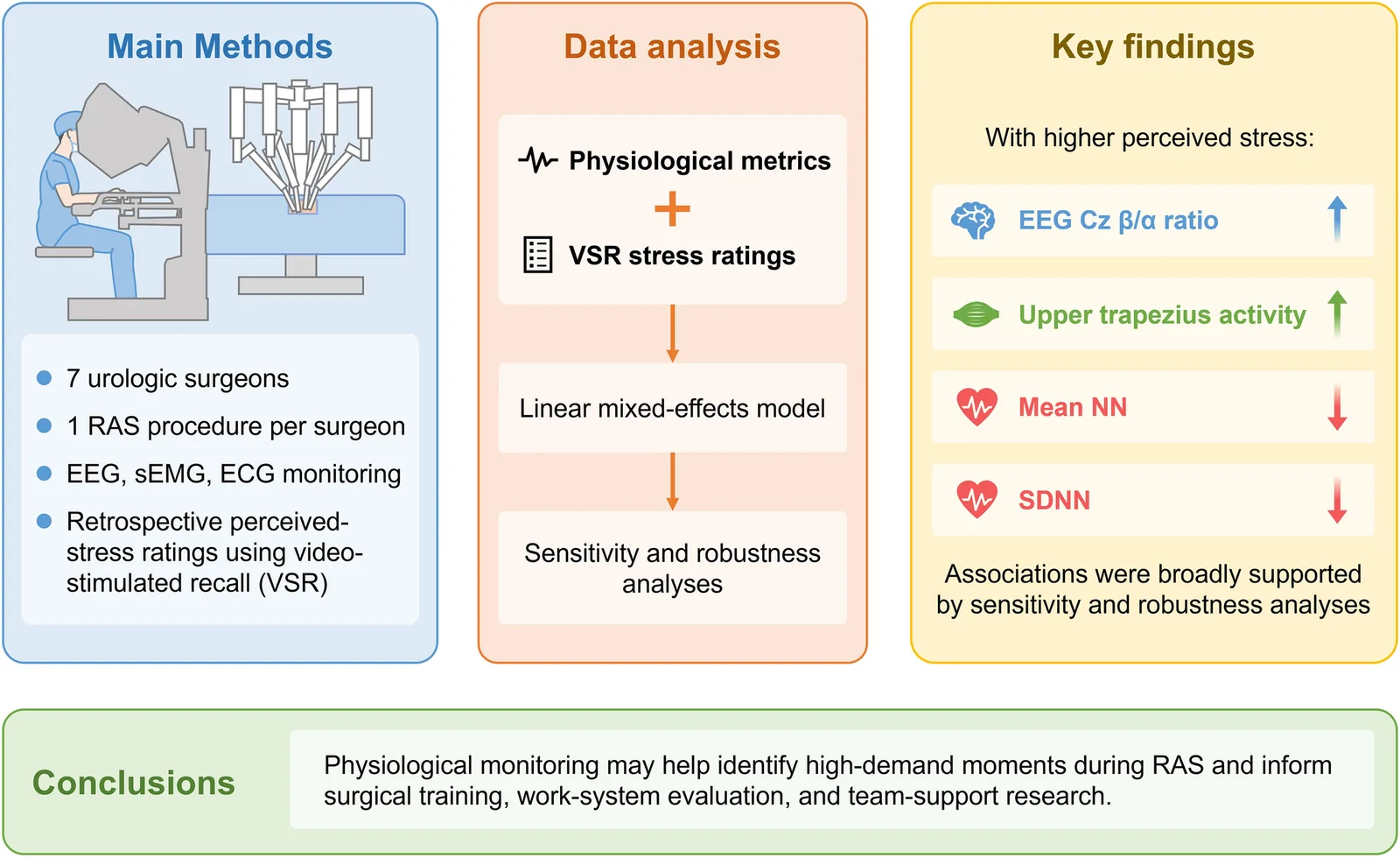

Robot-assisted surgery (RAS), while offering superior precision and dexterity, imposes a unique cognitive and psychomotor burden on the surgeon [1, 2]. The complexity of these procedures requires sustained vigilance and fine motor regulation, amplifying the impact of surgeon state on surgical safety [3]. In this context, stress is an expected feature of live surgery. However, excessive or poorly regulated stress may affect attention, motor control, and team communication, with potential consequences for surgical performance and safety [4, 5].

We define perceived stress as the surgeon’s subjective response to momentary mismatches between task demands and available coping resources [6, 7]. Distinct from cumulative mental workload, it is a dynamic state with measurable psychophysiological correlates. Stress engages multiple physiological subsystems, including cortical processing, autonomic regulation, and neuromuscular control [8, 9]. Reliance on any single physiological proxy may provide only a partial account, whereas multimodal measurement may offer a more complete characterization of perceived stress across complementary physiological domains [10, 11].

An important limitation in prior work is the reliance on simulations, which do not fully capture the ecological context of real surgery, including environmental distractions, clinical stakes, and surgical teamwork that define the actual intraoperative experience. In addition, commonly used subjective references such as NASA-TLX and SURG-TLX are often collected post-operatively as a single summary score, providing limited capacity for capturing intraoperative fluctuations [12, 13]. Moreover, existing intraoperative research has often relied on segmenting data by surgical phases [14–16]. Such approaches may average out critical short-term fluctuations and may not fully capture stress-related variation at a finer temporal scale. As a result, current literature lacks robust evidence on which physiological metrics consistently track perceived stress across surgeons during live RAS.

These challenges are particularly salient in RAS, where the console surgeon must maintain prolonged visuomotor attention and fine motor control under real clinical stakes, yet short-term stress fluctuations may be less apparent to the surrounding team. Accordingly, a compact and workflow-compatible approach to intraoperative stress assessment may be especially useful in this setting.

To address these challenges, we conducted a field study of live RAS using multimodal physiological recordings and retrospective stress ratings obtained by video-stimulated recall (VSR). We used hierarchical mixed-effects models to examine which physiological metrics were consistently associated with perceived stress across surgeons, and performed robustness and sensitivity analyses to assess the stability of the observed associations under realistic operating room (OR) constraints.

## Materials and methods

This was a prospective observational field study conducted at Chiba University Hospital, Chiba, Japan. Data collection was conducted between December 2024 and February 2025. The manuscript was prepared in accordance with the Strengthening the Reporting of Observational Studies in Epidemiology (STROBE) guideline for cohort studies.

### Participants

Surgeon participants were recruited at Chiba University Hospital. Eligible participants were urologic surgeons who served as the primary console surgeon for a RAS procedure during the study period and had prior robotic console experience of at least 10 cases.

Eligible surgeons were identified from the Department of Urology surgical schedule and approached before the procedure. Enrollment required written informed consent from both the surgeon and the patient. For each enrolled surgeon, one eligible elective RAS procedure was included. The included procedure was not necessarily the only RAS procedure performed by that surgeon during the study period. When more than one eligible procedure was available for the same surgeon, the first procedure for which surgeon and patient consent and complete multimodal recording arrangements were in place before surgery was included. Procedure inclusion was not based on procedure type or anticipated operative difficulty. Cases were excluded from the final analysis if the recorded physiological data were of insufficient quality for the prespecified analyses.

The study protocol was approved by the Chiba University Hospital Observational Research Ethics Review Committee (Approval No.: HK202407-01) and was conducted in accordance with the Declaration of Helsinki. Written informed consent was obtained from all participating surgeons and patients before data collection. All data were de-identified and stored in encrypted form, and participants retained the right to withdraw at any time.

### Data acquisition

**Fig. 1:**
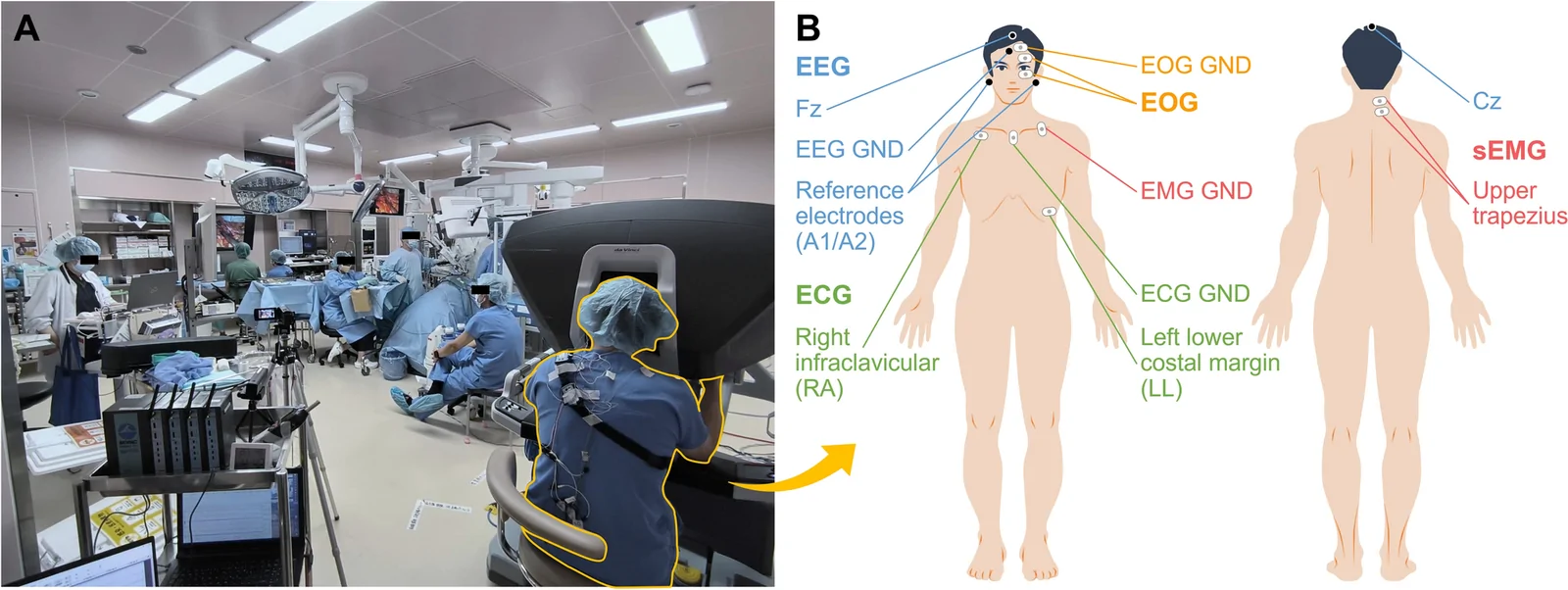
Intraoperative recording setup and electrode placement. (**A**) Real-world robot-assisted surgery (RAS) setup during intraoperative physiological recording. (**B**) Electrode placement for physiological recording. *EEG* electroencephalography, *EOG* electrooculography, *ECG* electrocardiography, *sEMG* surface electromyography, *GND* ground, *RA* right-arm ECG lead (placed at the right infraclavicular site), *LL* left-leg ECG lead (placed at the left lower costal margin)

Data were collected intraoperatively during RAS procedures using the da Vinci Xi Surgical System (Intuitive Surgical, Inc., Sunnyvale, CA, USA). The intraoperative recording setup and electrode placement are shown in Fig. 1. Multimodal physiological signals, including electroencephalography (EEG), electrooculography (EOG) for ocular-artifact correction, surface electromyography (sEMG), and electrocardiography (ECG), were synchronously recorded. Sound level near the surgeon console was also logged as a potential contextual covariate.

Physiological signals were sampled at 1000 Hz using a BIOPAC MP160 system with BioNomadix wireless transmitters and recorded via AcqKnowledge (v5.0) software (BIOPAC Systems, Inc., Goleta, CA, USA). Sound levels near the surgeon console were logged in A-weighted decibels (dB(A)) at 1-s intervals using a digital sound level meter (SLM-25, Gain Express Holdings Ltd.) set to FAST time weighting and oriented toward the surgeon console.

EEG was recorded from the frontal midline (Fz) and central midline (Cz) scalp locations according to the international 10–20 system, referenced to linked earlobes (A1/A2), with a forehead ground. Vertical EOG was measured in a bipolar montage with electrodes placed above and below the left eye. Bipolar sEMG was acquired from the right upper trapezius region, with electrodes placed closer to the posterior cervical midline to reduce the potential influence of upper-limb movements during console operation. ECG was recorded using a modified Lead II configuration with three chest electrodes placed at the right infraclavicular site, manubrium ground, and left lower costal margin.

The selection of monitoring modalities was informed by a previous pilot evaluation in a simulated RAS environment [17] and assessed for potential interference with console operation and OR workflow in consultation with the surgical members of the study team, including a senior surgeon certified as a proctor for robot-assisted urological surgery by the Japanese Urological Association and the Japanese Society of Endourology and Robotics. All sensors and electrodes were attached before the procedure, with cable routing and recording-device placement arranged to avoid interference. No procedure required interruption or modification due to the monitoring setup.

### Stress annotation and data windowing

**Fig. 2:**
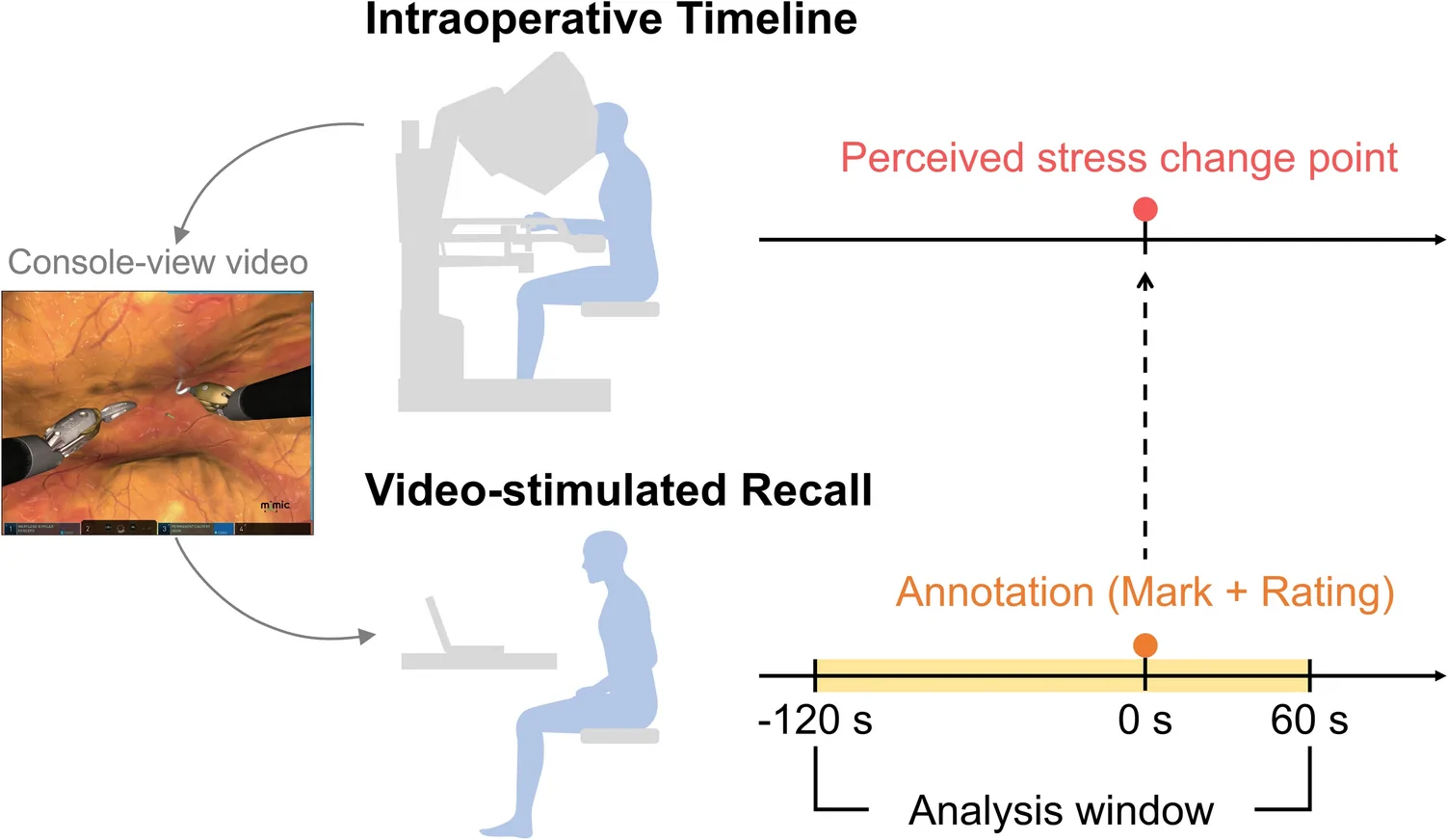
Video-stimulated recall-based stress annotation and analysis-window definition. Surgeons reviewed the console-view video, marked perceived stress change points, and assigned stress ratings. A 180-s analysis window was defined from − 120 s to + 60 s relative to each marked time point. The console-view image shown in this figure is a simulator image and does not depict an included patient procedure

To avoid disrupting the surgical workflow, perceived stress was assessed retrospectively using a VSR protocol [18–20]. Participating surgeons provided stress annotations while reviewing their respective console-view videos at the earliest feasible time given their clinical commitments, with all sessions completed within 5 days post-surgery [21]. For each annotation, surgeons marked the moment at which they perceived a transition into a different stress state and then rated the corresponding stress level on a 10-point numeric rating scale (NRS; 0 = no stress, 9 = maximum stress). The VSR-based annotation workflow and analysis-window definition are illustrated in Fig. 2.

A 180-s analysis window spanning − 120 s to + 60 s relative to the marked transition time was extracted for each annotation. This asymmetric window was used because retrospectively identified transition times may not align exactly with the onset of the associated physiological changes; the longer pre-transition segment was intended to capture preceding physiological build-up while retaining activity close to the reported transition.

### Signal preprocessing

**Fig. 3:**
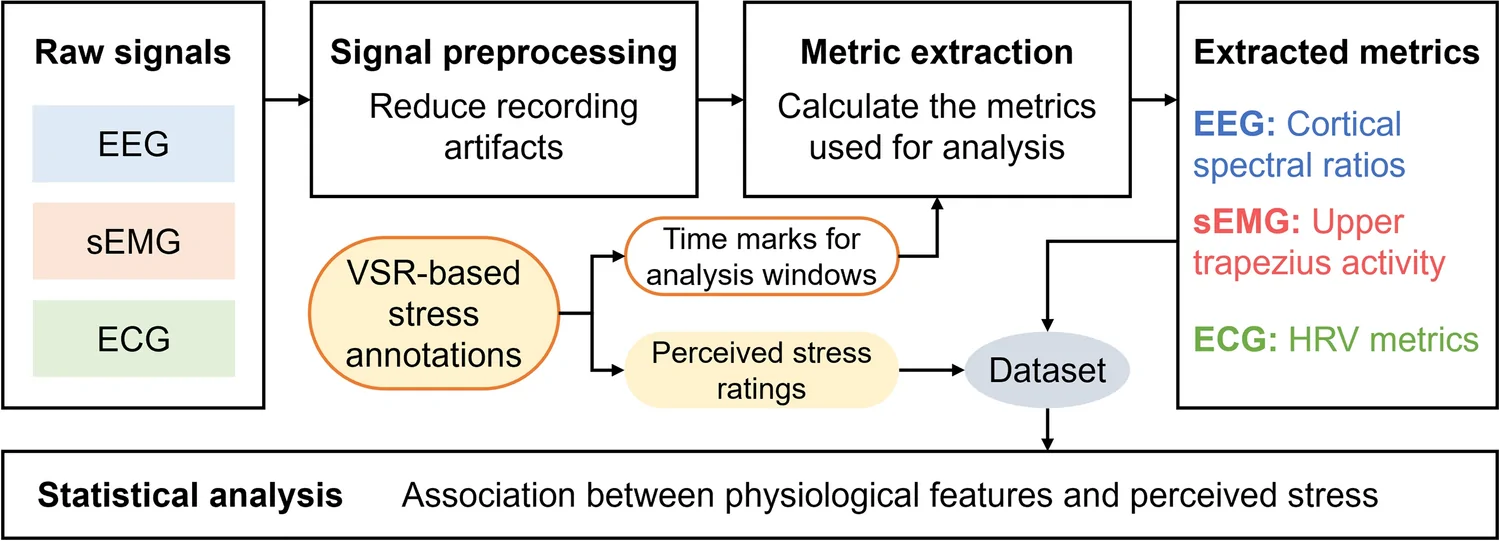
Signal processing and analysis dataset construction. Raw physiological signals were preprocessed, metrics were extracted within VSR-defined analysis windows, and the resulting physiological features were combined with perceived stress ratings for statistical analysis. *EEG* electroencephalography; *ECG* electrocardiography; *sEMG* surface electromyography; *HRV* heart rate variability; *VSR* video-stimulated recall

Figure 3 provides an overview of the preprocessing, metric extraction, and VSR-based annotation-matching workflow used to generate the final analysis dataset. Signal preprocessing was conducted in AcqKnowledge (v5.0). Preprocessing was performed to reduce common recording artifacts and improve the reliability of the physiological metrics. Because the main sources of noise differ across EEG, sEMG, and ECG, modality-specific preprocessing steps were applied.

For EEG, ocular activity such as eye movements and blinking was corrected using the simultaneously recorded EOG signal. The EOG signal was first bandpass-filtered at 0.1–6 Hz to isolate low-frequency ocular artifacts, and its contribution was then regressed out from the EEG signal. The corrected EEG signal was subsequently bandpass-filtered at 1–40 Hz to retain the frequency range used for the EEG spectral analysis while reducing slow drift and high-frequency noise [22].

For sEMG, the signal was bandpass-filtered at 20–450 Hz to retain muscle-related electrical activity and reduce low-frequency movement artifacts and high-frequency noise [23]. A 50-Hz notch filter was also applied to remove power-line interference.

For ECG, the signal was bandpass-filtered at 0.5–35 Hz to facilitate reliable detection of cardiac R peaks. R peaks were detected from the filtered ECG signal, and the resulting R–R interval series was visually inspected for artifacts. When erroneous intervals were identified, the waveform detection thresholds were adjusted to improve R-peak identification. This procedure yielded a verified normal-to-normal (NN) interval series for heart rate variability (HRV) analysis [24].

### Metrics calculation

Metrics calculation was conducted in Python (v3.11) using SciPy, NumPy, and pandas libraries [25–27]. All metrics were computed within the 180-s windows defined for each annotation. Annotations with insufficient data duration at the recording margins were excluded from the analysis. For ratio, spectral-power, and amplitude metrics, natural-log transformation, denoted as ln in the metric names, was applied to reduce right skewness before statistical analysis. The extracted metrics are summarized in Table 1.

**Table 1:** Multimodal physiological metrics included in the analysis of intraoperative stress during robot-assisted surgery (RAS)

Modality	Metric		Physiological significance
EEG	Fz ln(*θ*/*α*)		Cognitive workload/engagement
	Cz ln(*β*/*α*)		Cortical arousal/alertness
sEMG	Upper trapezius ln(mean RMS)		Muscle recruitment intensity and tonic tension
ECG	Time-domain HRV	Mean NN	Mean interbeat interval
		RMSSD	Parasympathetic activity
		SDNN	Global autonomic variability
	Frequency-domain HRV	ln(LF)	Mixed sympathetic and parasympathetic fluctuations
		ln(HF)	Parasympathetic modulation

*EEG* electroencephalography, *sEMG* surface electromyography, *ECG* electrocardiography, *ln* natural logarithm, *RMS* root mean square, *HRV* heart rate variability, *NN* normal-to-normal, *RMSSD* root mean square of successive differences, *SDNN* standard deviation of NN intervals, *LF* low-frequency, *HF* high-frequency

For EEG, power spectral density (PSD) was estimated for each analysis window using modified Welch’s method with 2-s segments, 50% overlap, and median averaging to reduce the influence of short, high-amplitude artifacts on the spectral estimate [28]. Band-power ratios were calculated as the natural logarithm of the theta-to-alpha ratio at Fz (ln(*θ*/*α*)) and the beta-to-alpha ratio at Cz (ln(*β*/*α*)), with frequency bands defined as theta (*θ*, 4–8 Hz), alpha (*α*, 8–13 Hz), and beta (*β*, 13–30 Hz).

For sEMG, the preprocessed signal in each 180-s analysis window was divided into overlapping 0.2-s subwindows with a step size of 0.05 s. The root mean square (RMS) amplitude was calculated for each subwindow because RMS provides a standard summary of EMG amplitude and was used here as an index of upper trapezius muscle activity. The sEMG metric was defined as the natural logarithm of the mean RMS across all subwindows within each analysis window [29].

For HRV, time-domain metrics were computed directly from the verified NN interval series within each 180-s analysis window. These included the Mean NN interval (Mean NN), the standard deviation of NN intervals (SDNN), and the root mean square of successive differences between adjacent NN intervals (RMSSD). For frequency-domain HRV analysis, the NN interval series was linearly interpolated and resampled at 4 Hz because spectral analysis requires an evenly sampled time series. Welch’s method was then applied using 64-s Hann-windowed segments with 50% overlap and constant detrending to estimate low-frequency power (LF, 0.04–0.15 Hz) and high-frequency power (HF, 0.15–0.40 Hz), and the resulting LF and HF powers were natural-log transformed [24].

For console-area sound level, the equivalent continuous A-weighted sound level (LAeq) was calculated for each 180-s analysis window from the A-weighted sound-level values logged at 1-s intervals, using the standard energy-averaging definition [30].

### Statistical analysis

Statistical analyses were conducted in R (v4.4.2) utilizing lme4, nlme, brms, and mgcv [31–33]. Hierarchical modeling and supplementary robustness analyses were used to examine the relationship between physiological metrics and surgeon-perceived stress. Before modeling, physiological metrics were standardized within each surgeon by mean-centering and scaling by the pooled within-surgeon standard deviation. This expressed each value relative to that surgeon’s own usual physiological level and allowed the analysis to focus on within-surgeon changes rather than baseline differences between surgeons. For refitting and resampling analyses, standardization parameters were recomputed within each subset to prevent data leakage. Because directly transferable prior effect-size estimates for this specific multimodal field design were not sufficiently established, no formal a priori power calculation was performed. For frequentist analyses, two-sided *P* < 0.05 was considered statistically significant.

#### Primary association analysis

The primary analysis used a linear mixed-effects model (LMM) to estimate the association between physiological metrics and perceived stress. This model was selected because multiple annotated windows were obtained from each surgeon, and observations from the same surgeon cannot be treated as fully independent. A surgeon-level random intercept was included to allow each surgeon to have a different baseline stress-rating level, while estimating the overall association between physiological metrics and perceived stress across the dataset.

Models allowing the physiological associations to vary by surgeon through random slopes were explored, but these more complex models were not sufficiently stable given the small number of surgeons and the number of annotated windows per surgeon. Therefore, the primary model used a more parsimonious random-intercept specification. All computed physiological metrics were entered as fixed effects. Multicollinearity among predictors was assessed using variance inflation factors (VIF), with all values remaining below 5.

Because stress annotations obtained close together within the same surgical case may be more similar than annotations separated by longer intervals, we examined whether the primary model needed to account for time-related correlation between residuals. In addition to the model treating residuals as independent after accounting for surgeon, we attempted to fit a continuous-time first-order autoregressive structure (CAR(1)) to account for possible temporal dependence among unevenly spaced annotations.

#### Robustness and sensitivity analyses

Given the small number of participants and the inherent variability of retrospective ratings, five supplementary analyses were performed to evaluate robustness and sensitivity.

Ordinal validation: Although NRS scores are commonly treated as approximately continuous in applied analyses, the 0–9 stress ratings are formally ordered categories [34, 35]. In this small field dataset, with a limited number of participant surgeons and potentially uneven use of score levels, the findings could be more sensitive to this modeling assumption. We therefore performed an ordinal validation analysis to test whether the main directional findings were preserved when stress ratings were modeled as ordered categories rather than as continuous scores. We fitted a Bayesian cumulative-logit mixed model, a standard ordinal regression model for ordered outcomes, using the same physiological predictors as in the primary analysis and a random intercept for surgeon. Weakly informative priors were used to stabilize estimation, and posterior summaries are reported as medians with 95% highest density intervals (HDIs).

Leave-one-surgeon-out (LOSO) refitting: Because the dataset included a small number of surgeons, an apparent association could be disproportionately influenced by a single participant. To assess this possibility, the primary model was iteratively refitted while excluding one surgeon at a time. This analysis evaluated whether the main directional associations remained stable when any individual surgeon was removed.

Pairwise concordance: To provide an intuitive within-surgeon summary of the primary model, we examined whether operative moments rated as more stressful by the same surgeon also had higher fitted values from the primary model. For each surgeon, pairs of annotated windows with different stress ratings were compared. When one window had a higher stress rating than another, we assessed whether the model also assigned a higher fitted value to that window. This pairwise agreement was summarized using the concordance index (C-index), calculated from fixed-effect-only fitted values from the final primary LMM for within-surgeon annotation pairs with rating differences of |ΔScore| ≥ 1 and |ΔScore| ≥ 2. Surgeon-specific C-index values were then averaged across surgeons, with 95% CIs estimated by surgeon-level bootstrap resampling.

Non-overlapping subset analysis: Because some 180-s analysis windows overlapped in time and could therefore include shared physiological data, the primary LMM was refitted using a strict subset of non-overlapping annotation windows. This tested whether the main associations remained stable after removing window overlap.

Control for local acoustic activity: Sound around the console may serve as a coarse indicator of local operative activity, including team communication, device sounds, alarms, or other environmental noise. Because such activity may coincide with demanding operative moments and could be related to both perceived stress and physiological responses, LAeq was added as a covariate to the primary LMM to examine whether the main physiological associations remained stable after accounting for local sound level.

## Results

**Fig. 4:**
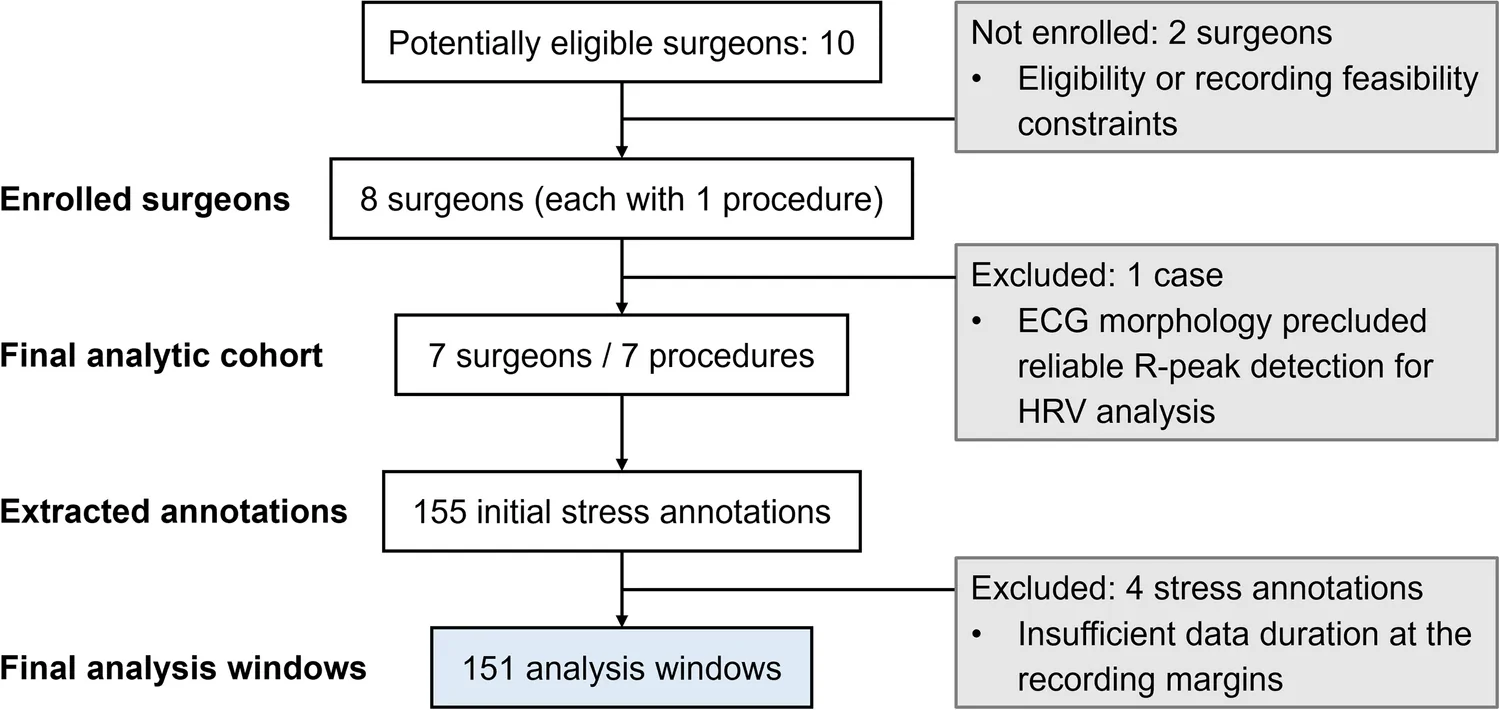
Flow diagram of participant, procedure, and analysis-window inclusion. *RAS* robot-assisted surgery; *ECG* electrocardiography; *HRV* heart rate variability

**Fig. 5:**
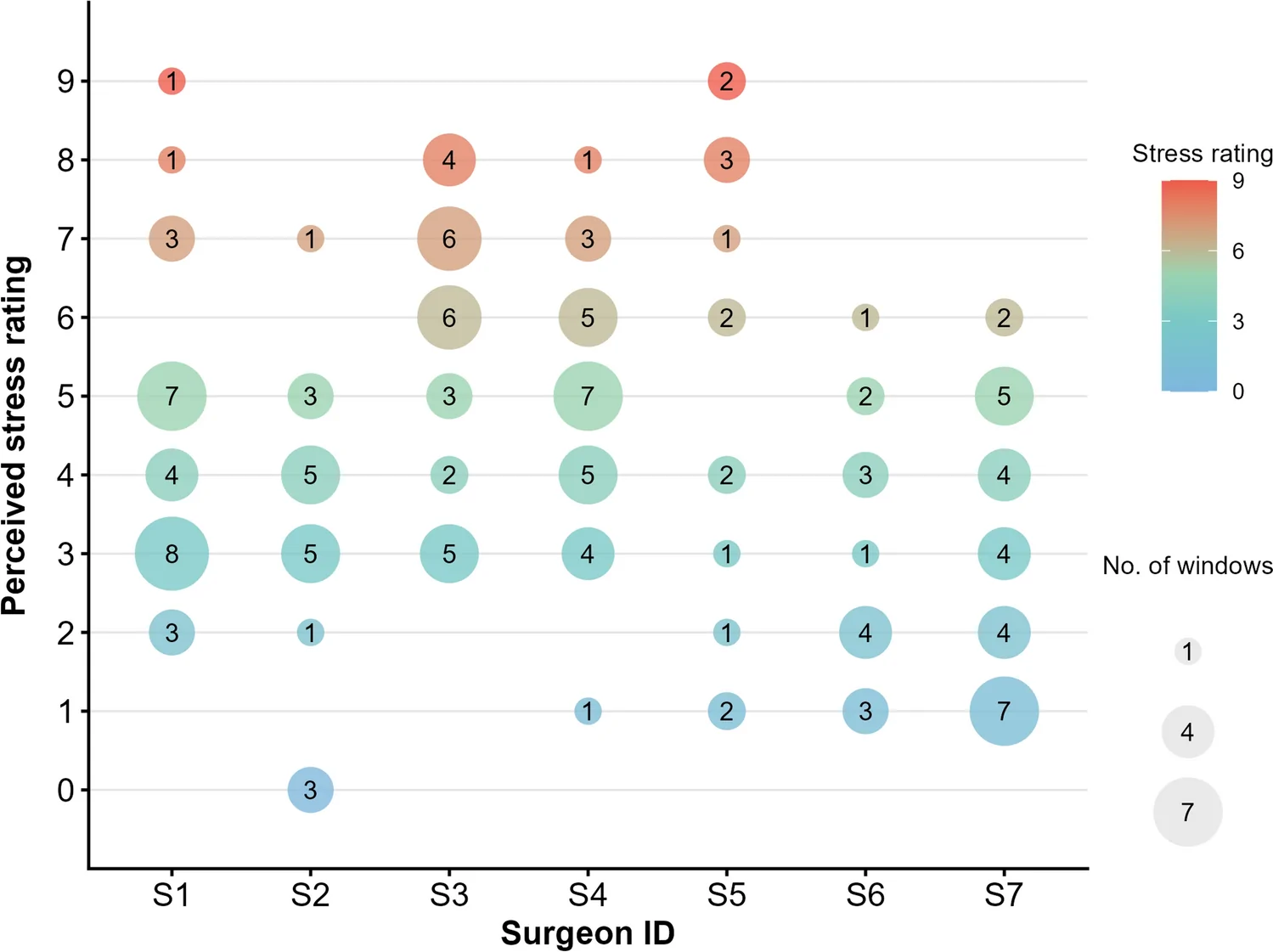
Distribution of analyzed windows across surgeons and perceived-stress ratings. Each bubble represents the number of analyzed windows for a given surgeon and perceived-stress rating. Bubble size and the number inside each bubble indicate the number of windows. Color indicates the perceived-stress rating (Color figure online)

As illustrated in Fig. 4, 10 urologic surgeons with prior robotic console experience of at least 10 cases were identified as potential participants during the study period; eight were enrolled, each contributing data from one RAS procedure. One case was excluded because ECG morphology precluded reliable R-peak detection and the planned HRV analysis. The final analyzed cohort comprised seven male senior urologists (mean age: 40.4 ± 6.0 years), with post-residency experience ranging from 8 to 24 years and cumulative robotic case volumes ranging from approximately 10 to 500 procedures. Table 2 summarizes surgeon-level characteristics, procedure type, and the number of analyzed windows per surgeon. All analyzed procedures were elective and included three robot-assisted radical prostatectomies, three robot-assisted partial nephrectomies, and one robot-assisted adrenalectomy. Of 155 initial annotations, four were excluded because of insufficient data duration at the recording margins, yielding 151 final analysis windows (14–27 per surgeon). The distribution of analyzed windows across surgeons and perceived stress ratings is shown in Fig. 5.

**Table 2:** Surgeon-level characteristics, procedure type, and analyzed windows

Surgeon ID	Post-residency experience, years	Cumulative robotic case volume, *n*	Procedure type	Analyzed windows, *n*
S1	14	~ 20	Robot-assisted radical prostatectomy	27
S2	8	~ 25	Robot-assisted radical prostatectomy	18
S3	24	~ 250	Robot-assisted adrenalectomy	26
S4	11	~ 50	Robot-assisted partial nephrectomy	26
S5	8	~ 10	Robot-assisted partial nephrectomy	14
S6	21	~ 200	Robot-assisted radical prostatectomy	14
S7	18	~ 500	Robot-assisted partial nephrectomy	26

### Primary model results

**Fig. 6:**
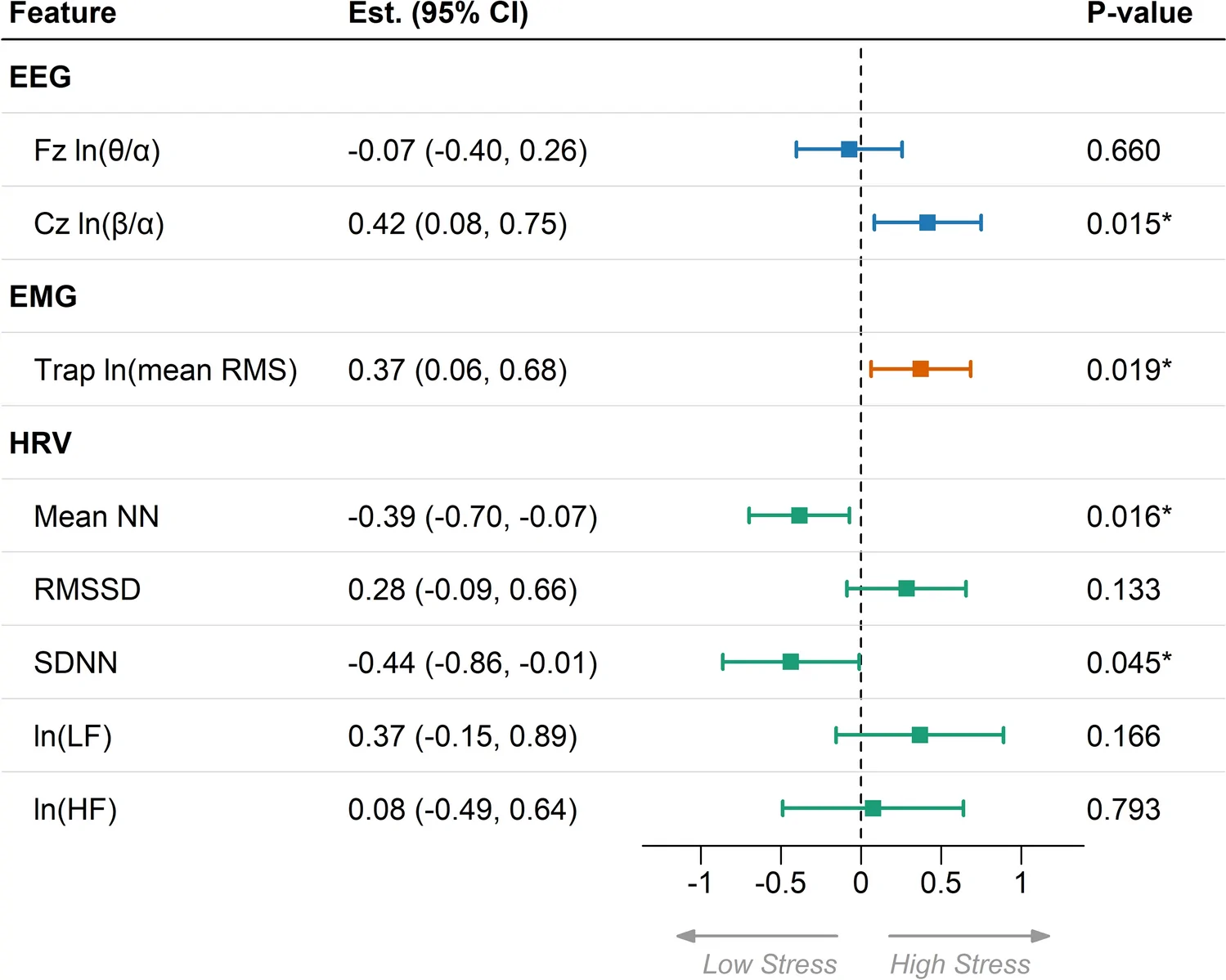
Primary model fixed-effect coefficients (*β*) with 95% confidence intervals (CIs). Positive *β* values denote higher perceived stress. *EEG* electroencephalography, *sEMG* surface electromyography, *HRV* heart rate variability, *ln* natural logarithm, *Trap* trapezius, *RMS* root mean square, *NN* normal-to-normal, *RMSSD* root mean square of successive differences, *SDNN* standard deviation of NN intervals, *LF* low-frequency, *HF* high-frequency

Figure 6 presents the fixed-effect estimates from the random-intercept LMM assuming independent residuals, which was used for the primary analysis. The model with a CAR(1) residual-correlation structure failed to converge and was therefore not considered further. Four physiological metrics showed statistically significant associations with perceived stress. For EEG, the Cz ln(*β*/*α*) ratio was positively associated with stress ratings (*β* = 0.42, 95% CI 0.08 to 0.75, *P* = 0.015). For sEMG, upper trapezius ln(mean RMS) was positively associated with stress (*β* = 0.37, 95% CI 0.06 to 0.68, *P* = 0.019). For HRV, Mean NN was negatively associated with stress (*β* = − 0.39, 95% CI − 0.70 to − 0.07, *P* = 0.016). SDNN was significantly negatively associated with perceived stress (*β* = − 0.44, 95% CI − 0.86 to − 0.01, *P* = 0.045). Other metrics, including RMSSD, the frequency-domain HRV metrics ln(LF) and ln(HF), and Fz ln(*θ*/*α*), were not significantly associated with perceived stress.

**Fig. 7:**
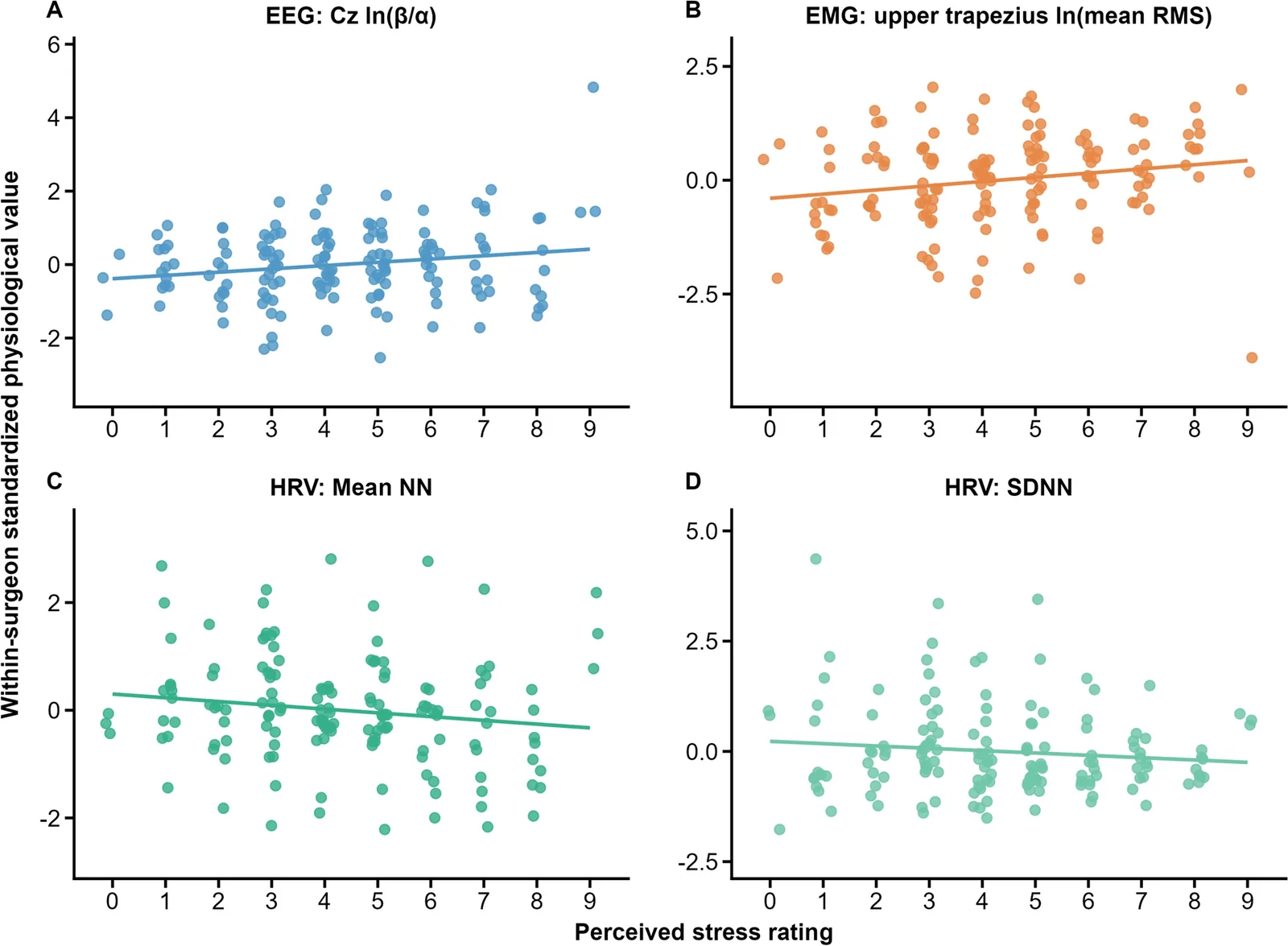
Observed distributions of physiological metrics significantly associated with perceived stress. **A** EEG Cz ln(*β*/*α*), **B** sEMG upper trapezius ln(mean RMS), **C** HRV Mean NN, and **D** HRV SDNN. Points represent within-surgeon standardized analysis windows; lines indicate descriptive linear trends. *EEG* electroencephalography, *sEMG* surface electromyography, *HRV* heart rate variability, *ln* natural logarithm, *RMS* root mean square, *NN* normal-to-normal, *SDNN* standard deviation of NN intervals

To aid interpretation of the primary mixed-effects model, Fig. 7 shows the observed distributions of the four physiological metrics that were significantly associated with perceived stress. These plots provide descriptive visualizations of the within-surgeon standardized values across stress ratings. Cz ln(*β*/*α*) and upper trapezius ln(mean RMS) showed upward descriptive trends with higher perceived-stress ratings, whereas Mean NN and SDNN showed downward descriptive trends. These visual patterns were consistent with the directions of the primary model estimates.

### Robustness and sensitivity findings

#### Ordinal validation

**Fig. 8:**
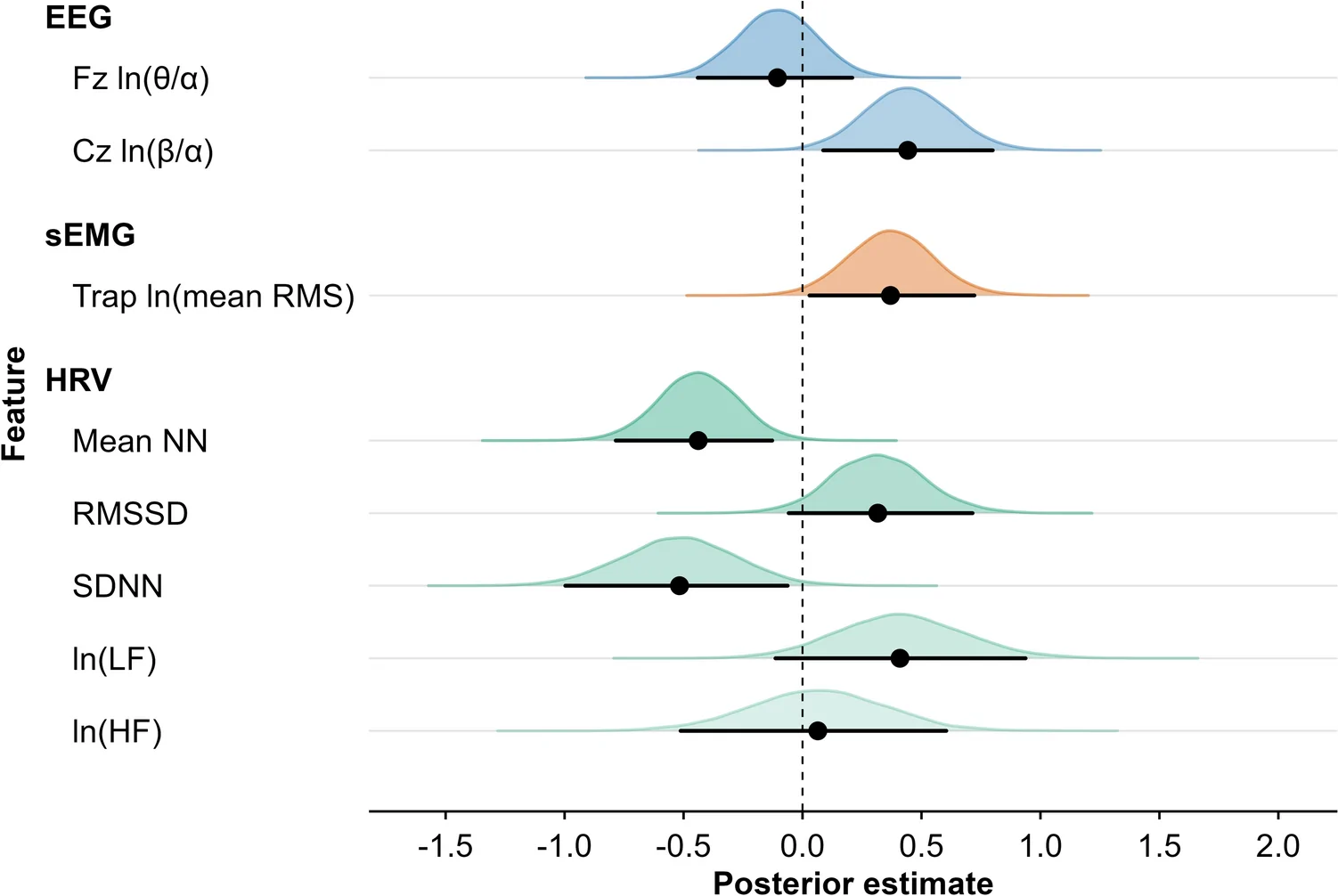
Bayesian ordinal regression of perceived stress. Dots and bars denote posterior medians and 95% highest density intervals (HDIs). *EEG* electroencephalography, *sEMG* surface electromyography, *HRV* heart rate variability, *ln* natural logarithm, *Trap* trapezius, *RMS* root mean square, *NN* normal-to-normal, *RMSSD* root mean square of successive differences, *SDNN* standard deviation of NN intervals, *LF* low-frequency, *HF* high-frequency

The Bayesian cumulative-logit mixed model generally supported the directional pattern of the primary findings (Fig. 8). Posterior medians and 95% HDIs showed consistent directional support for four indicators, whose HDIs excluded zero: Cz ln(*β*/*α*) (*β* = 0.44, 95% HDI 0.09 to 0.80), upper trapezius ln(mean RMS) (*β* = 0.37, 95% HDI 0.03 to 0.72), Mean NN (*β* = − 0.44, 95% HDI − 0.79 to − 0.13), and SDNN (*β* = − 0.52, 95% HDI − 1.00 to − 0.06). The remaining indicators showed greater posterior uncertainty, with 95% HDIs overlapping zero.

#### LOSO refitting

**Fig. 9:**
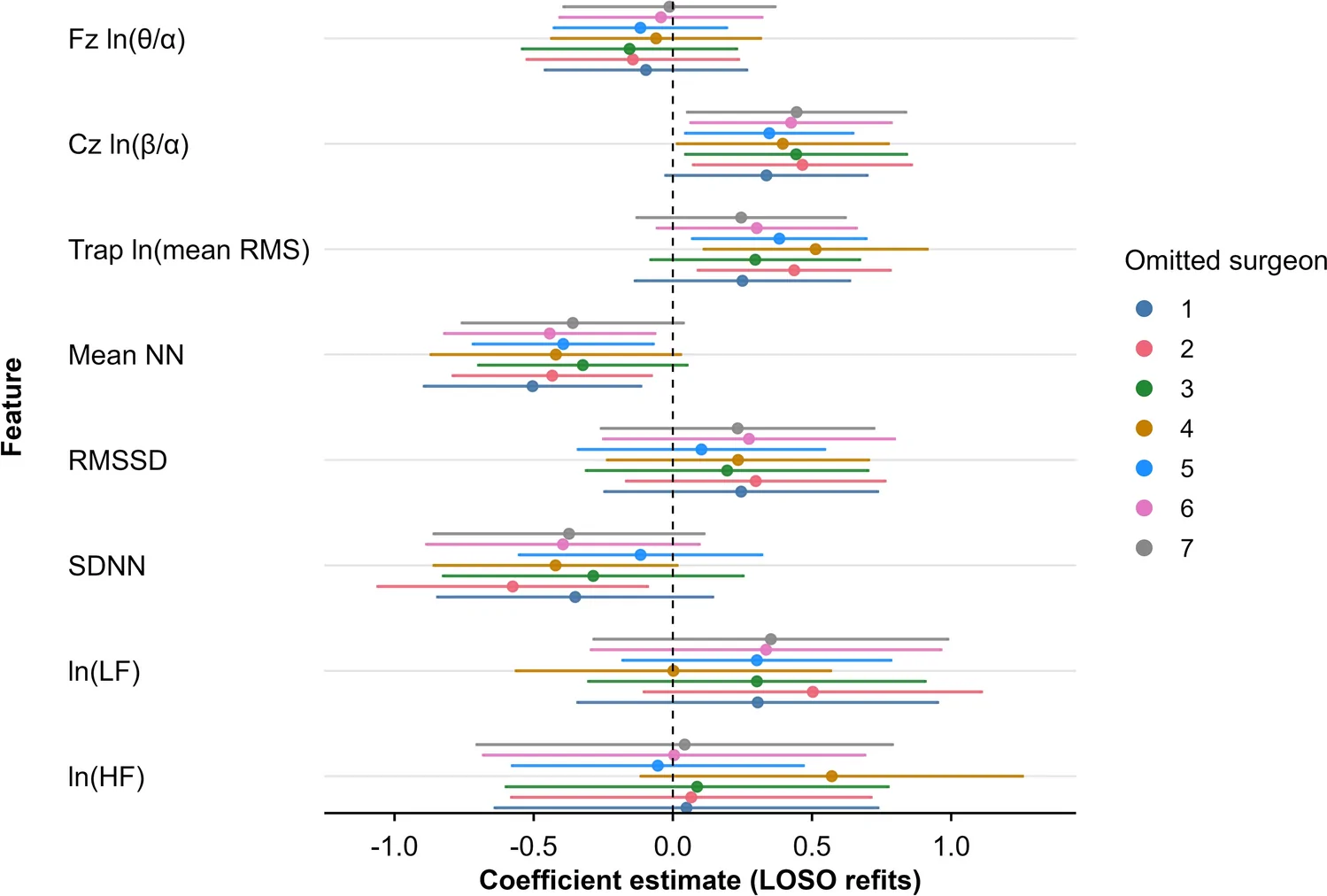
Leave-one-surgeon-out (LOSO) refitting. The primary model was refitted while omitting one surgeon at a time. *EEG* electroencephalography, *sEMG* surface electromyography, *HRV* heart rate variability, *ln* natural logarithm, *Trap* trapezius, *RMS* root mean square, *NN* normal-to-normal, *RMSSD* root mean square of successive differences, *SDNN* standard deviation of NN intervals, *LF* low-frequency, *HF* high-frequency

Across all LOSO iterations, Cz ln(*β*/*α*) and upper trapezius ln(mean RMS) remained positively signed, whereas Mean NN and SDNN remained negatively signed in relation to perceived stress (Fig. 9). Effect sizes varied only modestly across iterations (Cz ln(*β*/*α*): 0.34–0.47; upper trapezius ln(mean RMS): 0.25–0.51; Mean NN: − 0.50 to − 0.32; SDNN: − 0.58 to − 0.12), indicating that the overall directional patterns were not driven by any single participant.

#### Pairwise concordance

The overall C-index was 0.706 (95% CI 0.657–0.751) for annotation pairs with |ΔScore| ≥ 1 and 0.735 (95% CI 0.680–0.791) for pairs with |ΔScore| ≥ 2. These results indicate that, within the same surgeon, annotation windows rated as more stressful were generally assigned higher fitted values by the primary model.

#### Non-overlapping subset analysis

Overlapping segments accounted for 12.93% of total window time. In the strictly non-overlapping subset (*N* = 72), evidence for statistical significance was attenuated (Cz ln(*β*/*α*): *P* = 0.39; upper trapezius ln(mean RMS): *P* = 0.41; Mean NN: *P* = 0.11; SDNN: *P* = 0.09), but the estimated effects remained directionally consistent and of similar magnitude: Cz ln(*β*/*α*), primary *β* = 0.42 vs. pruned *β* = 0.34; upper trapezius ln(mean RMS), primary *β* = 0.37 vs. pruned *β* = 0.23; Mean NN, primary *β* = − 0.39 vs. pruned *β* = − 0.55; and SDNN, primary *β* = − 0.44 vs. pruned *β* = − 0.60. These findings suggest that the primary associations were not driven by window overlap.

#### Control for local acoustic activity

Including console-area sound level as a covariate did not materially alter the direction or statistical significance of the primary physiological associations. LAeq itself showed a positive but non-significant association with perceived stress (*P* = 0.107).

## Discussion

This study aimed to identify candidate physiological correlates of surgeon stress during live RAS under real-world OR constraints. We observed four indicators showing the most consistent within-surgeon associations with perceived stress: central EEG arousal (Cz ln(*β*/*α*)), upper trapezius muscle activation (ln(mean RMS)), and two HRV-derived indices of autonomic regulation (Mean NN and SDNN). Together, these findings support the value of a compact multimodal physiological profile for characterizing perceived stress in live RAS.

The positive association between Cz *β*/*α* and perceived stress is consistent with broader evidence linking the EEG *β*/*α* ratio to mental stress in high-demand settings [36, 37]. Lim et al., also conducting EEG measurement in live RAS, similarly identified intraoperative EEG correlates of mental state, though focusing on phase-level workload and using relative band power [15]. The absence of a reliable association for Fz *θ*/*α* suggests that perceived stress in this setting may not have primarily reflected sustained frontal cognitive-control demand, although frontal theta activity has been linked to cognitive control, anxiety, and uncertainty [38]. Upper trapezius sEMG also showed a positive association with perceived stress, consistent with evidence of sustained shoulder–neck muscular workload during robotic and laparoscopic procedures [39, 40]; together with psychophysiological evidence linking trapezius activation to stress-related tension, this suggests it reflects the overall burden of demanding operative moments without necessarily indexing a single stress-specific mechanism. For autonomic regulation, shorter Mean NN intervals and lower SDNN were associated with higher perceived stress, reflecting cardiac acceleration and reduced overall HRV. This direction is consistent with D’Ambrosia et al., who reported decreases in interbeat interval and SDNN during performance errors in RAS simulations [41]. It also aligns with Naiki et al., who used heart rate and estimated SDNN to assess mental stress during live RARP [16]. Although evidence from live RAS remains limited, HRV metrics have been widely used as objective physiological indices of surgeons’ mental stress across operative and simulation-based settings [42]. The weaker findings for RMSSD and frequency-domain metrics may reflect the limited reliability of short-window HRV estimation, particularly for frequency-domain metrics, as well as potential respiratory influences during high-precision maneuvers [43–46].

Capturing perceived stress during live surgery presents a challenge: intraoperative self-reports risk interrupting surgical workflow and may themselves induce stress, whereas postoperative questionnaires are susceptible to retrospective bias. To balance these constraints, the present study employed VSR, a method widely used in naturalistic research to help participants revisit the original perceptual context through video cues and reduce loss of detail. VSR methods have also been applied in surgical and OR research to examine otherwise difficult-to-observe cognitive processes. Chen et al. used VSR interviews to investigate factors influencing attending surgeons’ intraoperative guidance decision-making [21], while Dias et al. used a video-based recall protocol to elucidate cognitive processes among different team roles during cardiac surgery [18]. In RAS, the console-view video provides an intrinsic first-person perspective, enabling retrospective perceived stress annotations to be linked to specific operative moments while avoiding intraoperative self-reports that could interrupt the surgical workflow. However, these annotations remain retrospective and may be affected by recall-related imprecision, as discussed further in the “Limitations.”

Physiological recordings provide quantitative information on how surgeons respond to demanding operative moments. When combined with surgical video, instrument motion, and team communication, these measures could make postoperative review more specific by linking a technical event with the surgeon’s physiological response. For training feedback, this means that a difficult step can be examined in terms of response magnitude, duration, and recovery before the next demanding period, which retrospective description alone does not capture. Beyond individual case review, surgeon-state measures could serve as an outcome for evaluating the robotic surgery work system, including console ergonomics, communication routines, workload distribution, procedure-specific difficulty, and training stage; this is the more immediate practical application of the present approach. For intraoperative use, the most feasible role would be in supporting team coordination around the console surgeon’s workflow, such as minimizing avoidable distractions during high-demand periods, clarifying the next operative step, or identifying an appropriate pause point for a brief team update when the surgical situation allows. This is consistent with prior work suggesting that the timing of acute stress-management support may be important for surgical performance [47]. Existing structured-communication approaches aim to improve shared situational awareness within the OR team [48], and physiological state information could help future studies determine when such support is most useful, particularly during periods of sustained or recurrent demand.

## Limitations

This study has several limitations. First, the small, single-center cohort limited generalizability and prevented reliable modeling of procedure-specific effects or between-surgeon variability. Larger multi-center studies are needed to evaluate whether these associations are stable across surgeons, institutions, and procedure types. Second, VSR-based stress annotations were collected retrospectively within a maximum interval of 5 days and remained subject to recall-related imprecision and possible reconstruction bias inherent to retrospective self-report methods [49, 50]. Surgeons may have reorganized or reinterpreted their perceived stress after the event in light of their overall impression of how the case progressed. The small sample size limited our ability to formally evaluate the extent of these potential influences. Third, the intentionally minimal sensor configuration did not include complementary signals such as respiration or electrodermal activity (EDA), which could help disentangle affective arousal from physical exertion. Future integration of these measures, alongside instrument kinematics, may improve interpretability. Additionally, the LAeq covariate captured both local communication and surrounding OR sound. Future studies could employ distributed microphones or audio source separation techniques to more precisely isolate environmental acoustic stressors. Finally, this study identified physiological correlates of perceived stress but did not determine whether these measures predict technical errors, surgical performance, or patient outcomes. Prospective validation against objective performance and safety-related outcomes is required before these measures can be used for clinical decision support.

## Conclusions

In live RAS, higher VSR-based perceived stress reported by the console surgeon was associated with a higher central EEG *β*/*α* power ratio, greater upper trapezius sEMG activity, shorter Mean NN intervals, and lower SDNN. These associations were broadly supported by robustness and sensitivity analyses. The findings suggest that combining surgeon-reported stress annotations with multimodal physiological monitoring is feasible in RAS and may provide a basis for identifying high-demand operative moments during postoperative review, with potential applications in surgical training, work system evaluation, and team support research.
